# iRefR: an R package to manipulate the iRefIndex consolidated protein interaction database

**DOI:** 10.1186/1471-2105-12-455

**Published:** 2011-11-24

**Authors:** Antonio Mora, Ian M Donaldson

**Affiliations:** 1 Department for Molecular Biosciences, University of Oslo, P.O. Box 1041 Blindern, 0316 Oslo, Norway; 2 The Biotechnology Centre of Oslo, University of Oslo, P.O. Box 1125 Blindern, 0317 Oslo, Norway

## Abstract

**Background:**

The iRefIndex addresses the need to consolidate protein interaction data into a single uniform data resource. iRefR provides the user with access to this data source from an R environment.

**Results:**

The iRefR package includes tools for selecting specific subsets of interest from the iRefIndex by criteria such as organism, source database, experimental method, protein accessions and publication identifier. Data may be converted between three representations (MITAB, edgeList and graph) for use with other R packages such as igraph, graph and RBGL.

The user may choose between different methods for resolving redundancies in interaction data and how n-ary data is represented. In addition, we describe a function to identify binary interaction records that possibly represent protein complexes. We show that the user choice of data selection, redundancy resolution and n-ary data representation all have an impact on graphical analysis.

**Conclusions:**

The package allows the user to control how these issues are dealt with and communicate them via an R-script written using the iRefR package - this will facilitate communication of methods, reproducibility of network analyses and further modification and comparison of methods by researchers.

## Background

Currently, there are several protein interaction databases that are publicly available. iRefIndex is one effort to consolidate their information into one single repository while grouping redundant information [1]. This index allows the user to search for a protein and retrieve a non-redundant list of interactors for that protein along with all supporting experimental information. The primary interaction databases included in iRefIndex are BIND [2,3], BioGRID [4], CORUM [5], DIP [6], HPRD [7], IntAct [8], MINT [9], MPact [10], MPPI [11] and OPHID [12].

In this paper we introduce the iRefR package that allows the user to retrieve and work with the iRefIndex data set in an R environment [13]. In addition, we use iRefR to examine network properties for several organisms and ask how they are affected by the redundancy detection method and the n-ary data representation method chosen by the user.

First, we assemble interactomes based on two definitions of redundancy and assess how network properties change. The iRefIndex process assigns each protein interactor a hash-key that is based on the primary amino acid sequence and taxonomy identifier of the protein [1]. The key is called a "Redundant Object Group Identifier (ROGID)" and it can be used to group together identical protein interactors regardless of the database-accession system used to describe the protein in the original interaction record. The ROGIDs present in an interaction record are in turn used to create a key for the interaction record (RIGID: Redundant Interaction Group Identifier) that serves to group together all source records that share the same set of interactors [1].

The iRefIndex process also assigns a canonical ROGID (cROGID) to each protein interactor. This key serves to group together related proteins and interaction records that may be products of the same gene but that do not have the same sequence [14]. All members of a canonical group will have different ROGIDs but will share the same cROGID (the cROGID is identical to the ROGID of *one *of the group members that is chosen to represent the entire group of related proteins). A canonical RIGID (cRIGID) is constructed for each record from the cROGIDs and serves to group together records that describe the same set of proteins (at a canonical level).

Normalizing a network to its canonical form will have the effect of collapsing protein nodes and interaction edges that refer to the same gene products; as a result, the size of the network can be reduced and its network properties may be altered.

Second, we address the issue of how n-ary data representation alters network properties. N-ary interaction records (or so-called *complex data*) represent experimental evidence involving three or more proteins [14,15]. While binary data clearly supports an interaction between two proteins (A interacts with B), n-ary data only shows that a group of proteins were observed together in some experiment and the actual binary interactions between any given pair are either unknown or unspecified; for example, a list of proteins that are co-purified as members of a complex is an example of n-ary data. A third type of interaction data (polymer data) involve records with only one interactor type - these are records that describe two or more instances of the same molecule interacting with one another (e.g. homodimers). Source interaction records that contain three or more interactors can be identified and retrieved from iRefIndex using the iRefR package. By default, n-ary data is represented in the iRefIndex file using a "bipartite" model. Each member of the n-ary list is represented by a single edge between the list member and a single artificial entity that represents the group of proteins itself. These edges are not binary interactions but a pair wise representation of a list of proteins. This list-view of n-ary data is convenient for non-graphical applications, for example, over-representation analysis by comparison to other lists. Alternatively, these n-ary data may be converted to a "spoke model" representation where a set of edges is constructed between one chosen "hub" protein (in most cases the bait used in the experiment) and each of the other members of the n-ary list. This way, a group of *m *proteins will create *(m-1) *binary edges. A second alternative is a "matrix model" representation where every possible pairwise interaction among the members of the n-ary list is generated; this will create, m*(m-1)/2 edges. We compare graphical properties for several interactomes using these two methods to represent n-ary data or by leaving n-ary data out altogether.

We describe the iRefR package below. The user may select data according to source database, publication identifier, experimental method, and protein accession. The user may easily separate binary, n-ary and polymer interaction data. Summary statistics and network graphs may be constructed from any data subset using either the sequence-specific or canonical protein identifiers to resolve redundancies. N-ary data may be represented in a graphical format using either the spoke or matrix format. In addition, we describe a function to retrieve binary interaction records that may represent protein complexes (so-called spoke-represented complexes). Finally, iRefR is used together with other R graphical packages to assess the effect of the data consolidation method and n-ary data representation on some common statistical and graphical parameters.

## Results

### IRefR API Data Structures

**Figure 1:**
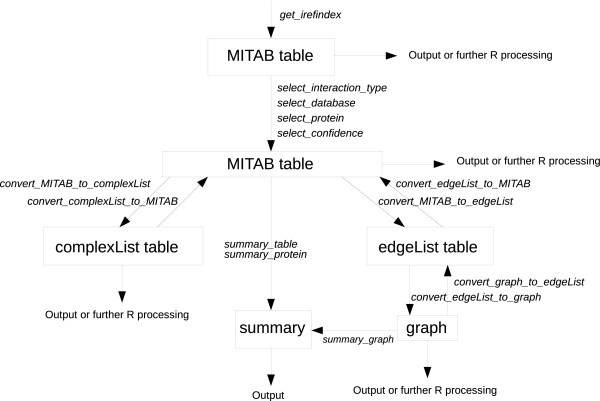
**Main functions and data types in iRefR**. iRefR uses four different data types (MITAB, complexList, edgeList and graph) and 17 functions to get the database, select a subset, convert between protein identifiers, convert between table formats, show statistics and make graphics. The figure shows 14 of the functions in relation to the main data types. All functions are listed and described in Table 1.

The iRefR application programming interface (API) is represented in Figure 1 as a set of data types that can be consumed and produced by various functions (see Table 1). The package is available from the Comprehensive R Archive Network http://cran.r-project.org/web/packages/iRefR/index.html. A tutorial is available as well as code used to generate data and figures for this paper (see Additional files 1 and 2). Function descriptions and examples are also available using the R help functionality (e.g., ?function_name).

**Table 1:** Functions in the iRefR package.

name	description
*get_irefindex*	Retrieve an iRefIndex data set by taxon identifier or release number as a data frame in MITAB format.
*create_id_conversion_table	Use an iRefIndex data set to generate a lookup table that can be used by *convert_protein_ID*.
*convert_protein_ID	Convert protein identifiers from one of the following formats: iROG ID, icROG ID, gene ID, RefSeq, PDB and UniProt, to any other protein identifier type in the list.
*select_interaction_type*	Select a subset of MITAB belonging to a certain interaction type (binary, polymer, complex).
*select_database*	Select (or exclude) a subset of MITAB belonging to one or a group of primary interaction databases.
*select_protein*	Select a subset of MITAB for a given protein identifier.
*select_confidence*	Select a subset of MITAB given a confidence or bibliometric score type and value (or value range).
*convert_MITAB_to_edgeList*	Convert MITAB data set to edgeList format. Can specify complex representation, directionality, node id type (iROG or icROG) and edge-weight.
*convert_edgeList_to_MITAB*	Retrieves subset of a MITAB data set matching an edgeList. User should specify node identifier type (iROG or icROG) used in edge list. Can specify interaction type to retrieve (binary, complex, polymer).
*convert_MITAB_to_complexList*	Convert MITAB data set to complexList format. Can specify node id type (iROG or icROG). Can specify if and how potential spoke-represented complexes should be included.
*convert_complexList_to_MITAB*	Retrieves subset of a MITAB data set matching a complexList. User should specify node identifier type (iROG or icROG) used in complex. Can specify whether potential spoke represented complex lines should be returned.
*merge_complexes_lists	Merge complexLists and remove duplicate complexes.
*convert_edgeList_to_graph*	Convert an edgeList table to a Graph object. Can specify if edges have directionality and graph package (graph/igraph) that will use the Graph object.
*convert_graph_to_edgeList*	Convert a Graph to an edgeList table.
*summary_protein*	Get Summary Information for a given Protein.
*summary_table*	Get Summary Information for a MITAB Table.
*summary_graph*	Get Summary Information for a Graph.

*Not shown in Figure 1. All other functions and their relation to input and output data types are shown in Figure 1.

There are three data table types used in the iRefR package: MITAB, complexList and edgeList. Graphs are an additional data type generated from edgeLists.

MITAB (molecular interaction tab-delimited) is a widely used format for distributing molecular interaction data as a tab-delimited file. It is based on the Human Proteome Organization Proteomics Standards Initiative Molecular Interactions (HUPO PSI-MI) format [16]. The use of this format is detailed on the iRefIndex wiki site [17]. The first 15 columns contain core information about the interaction, such as database accessions pointing to the interacting proteins and the experimental methods used to demonstrate the interaction.

The "complexList" data type is a table with two columns; the first column is a group identifier and the second column is a comma-separated list of protein identifiers that are all members of some n-ary interaction record. This representation has less information than the MITAB but simplifies working with groups of proteins, such as complexes; besides that, it has the advantage of allowing the treatment of n-ary data as vectors or lists, being amenable to statistical tests looking for significant overlap with other lists of genes or proteins.

Finally, the edgeList data type contains a list of edges (as pairs of protein identifiers) and their weights. N-ary interaction records may be converted to an edgeList using either a spoke, matrix or bipartite model approach. The edgeList format may be converted to a graph format that is consumable by functions in the igraph [18,19], or the graph [20] and RBGL [21] packages for R.

### IRefR API Functions

A single function called "get_irefindex" will retrieve the desired version of iRefIndex (7.0 or above) via File Transfer Protocol, either for all organisms or a specified organism and store it in an R data table in MITAB format (see Figure 1). The user may then use "select" functions to create a subset of the data based on a protein identifier (or list), the source database, publication identifiers, the interaction type (binary, complex or polymer) or a bibliometric score.

Data in MITAB format may be converted to the other data types (and back again) using the "convert" family of functions. The conversion of a MITAB file to a complexList or to an edgeList and a graph allows the user to choose an identifier type (canonical or not - see below) and a method to represent n-ary interaction data (spoke, matrix or bipartite).

iRefR uses the iRefIndex identifiers (either ROGIDs or canonical ROGIDs) to name proteins in the complexList, edgeList and graph data-structures. A function called "convert_protein_id" is provided to translate between ROGIDs, cROGIDs and the most commonly used protein identifiers such as RefSeq and UniProt accessions.

The iRefR package includes descriptive statistics functions for proteins and interaction data sets (see summary_protein, summary_table and summary_graph). "convert_edge_list_to_graph" allows the user to generate graphs that may be used by either of the two more used graphical packages in R ("igraph" [18] and "graph" [20]), for graph-theoretical analysis and manipulation.

A great deal of information can be retrieved or generated using iRefR. Such analyses and statistics can be updated every time the "iRefIndex" database is updated. As an example, the following studies were done using iRefR v.0.93 and iRefIndex v.8.0. The iRefR code used to generate the following tables and figures is included as Additional file 2.

### Interspecies Comparisons

**Figure 2:**
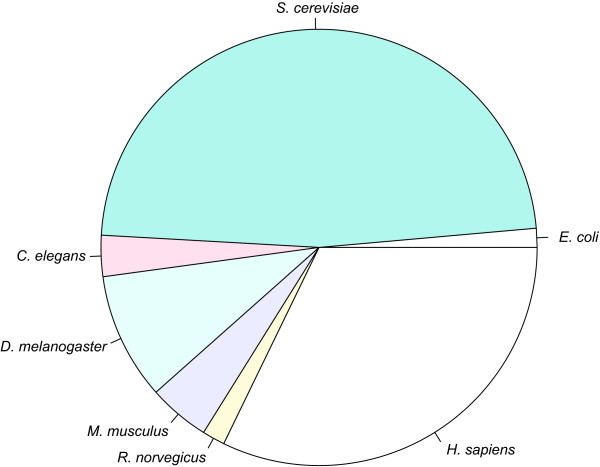
**Comparing number of interaction records available for seven. model organisms in iRefIndex**. *S.cerevisiae *has the best studied interactome to date (186513 distinct canonical interactions) followed by *H. sapiens *(125898 interactions), *D. melanogaster *(36773 interactions), *M. musculus *(17848), *C.elegans *(11905), *R. norvegicus *(6586) and *E.coli *(5500).

iRefR can be used to compare the sizes or graph-theoretical properties of seven different model organisms. Figure 2 shows an overview of the interactome data set sizes for these organisms. *S. cerevisiae *and *H. Sapiens *data constitute the bulk of the dataset, which is consistent with our previous observations [14,15].

These data may be broken down according to the type of interaction (binary, complex or polymer), the number of interactions in both canonical and non-canonical form, the number of interacting proteins in canonical and non-canonical form, and the statistics according to the primary database where the interaction has been taken from.

**Figure 3:**
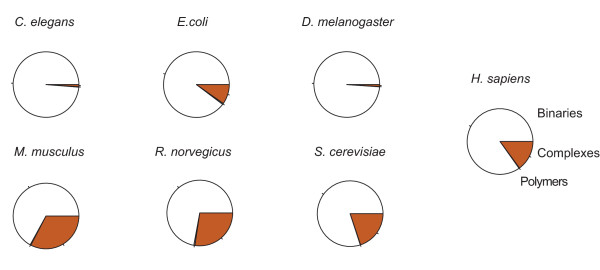
**iRefIndex Records per interaction type**. The figure shows how the ratio of binary:n-ary:polymer interaction records differ according to the organism. The binary:n-ary record ratio is **7:**1 for human, larger for most organisms and smaller for mice, rat and yeast. Polymer interaction records constitute a small fraction in all cases.

Figure 3 shows that the interaction databases are mainly formed by binary interactions. However, there are a significant number of n-ary records as well especially for human, mouse, rat and yeast. The human interactome is constituted by 19635 distinct proteins (non-canonical representation, i.e., isoforms counted as different proteins), that can be grouped in 17246 different canonical groups (isoforms included in one group) (Table 2). These proteins are involved in 119764 interactions (non-canonical representation), that are reduced to 107257 interactions when grouping isoforms into the same canonical groups of proteins (Table 3). Canonicalization has a less significant effect on the number of interactions for organisms where alternative splicing is absent as indicated by the ratio of canonical to non-canonical protein interactor counts (Table 2).

**Table 2:** Number of distinct proteins involved in protein-protein interactions in both non-canonical (ROGIDs) and canonical versions (cROGIDs).

organism	ROGIDs	ROGIDs -org	cROGIDs	cROGIDs -org	ratio
*C.elegans*	5942	5841	5475	5374	0.92
*D.melanogaster*	11676	11105	10027	9459	0.85
*E.coli*	2923	2673	2923	2673	1.00
*H.sapiens*	33180	19635	30690	17246	0.88
*M.musculus*	14280	7245	13714	6908	0.95
*R.norvegicus*	6337	2806	6176	2721	0.97
*S.cerevisiae*	13762	6332	13754	6329	1.00

ROGIDs: number of distinct proteins (sequence plus taxonomy id)

ROGIDs -org: number of distinct proteins of the specified organism

cROGIDs: number of distinct canonical groups of proteins

cROGIDs org: number of distinct canonical groups of proteins of the specified organism

ratio: ratio of cROGIDs-org to ROGIDs-org

**Table 3:** Number of distinct human interactions in both non-canonical (RIGIDs) and canonical versions (cRIGIDs).

dataset	RIGIDs	cRIGIDs
Complete Human Dataset	138570	125898
Human-Human Interactions	119764	107257
Binary Subset	132889	120425
Binary Human-Human Subset	114091	101792
Complex Subset	5232	5027
Complex Human-Human Subset	5226	5021
Polymer Subset	449	446
Polymer Human-Human Subset	447	444

iRefIndex has 138570 distinct human interactions (125898 after canonicalization), 119764 of which are human-human protein interactions (107257 after canonicalization). The majority of these data are binary records.

RIGID: number of distinct interactions (groups of distinct ROGIDs).

cRIGID: number of distinct interactions (groups of distinct canonical ROGIDs)

We also noted that interaction data sets for some organisms contain many interspecies interactions. For example, the *Homo sapiens *data set in Table 2 has 30690 distinct human proteins when interactions are retrieved that contain at least one human protein; however, there are only 17,246 distinct proteins when records are retrieved where all interactors are from human. These records may represent interactions between a human protein and a pathogenic species or cases where a protein from a second species has been listed as a substitute for a human protein. Analysts should be aware of this possibility and may wish to filter data accordingly.

### Regenerated complexes

**Figure 4:**
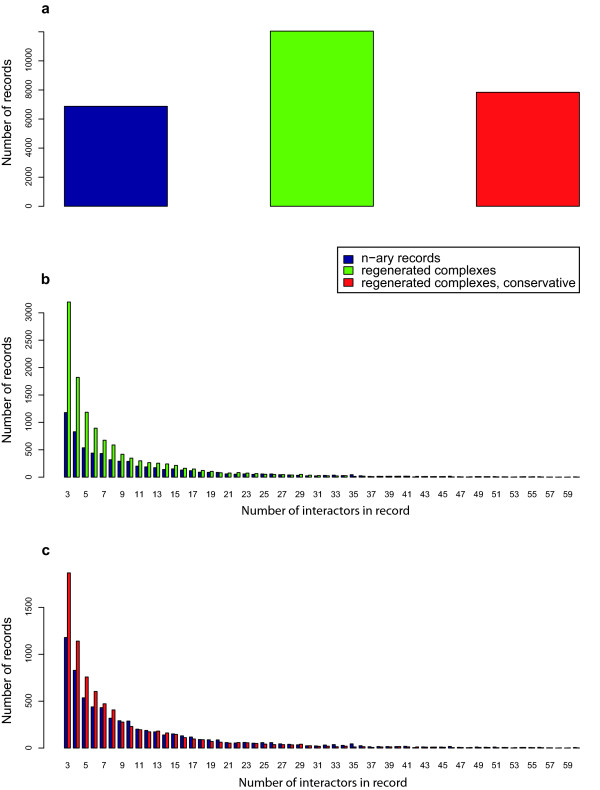
**Number and size of yeast n-ary records versus regenerated complexes**. **a**. Number of binary records in yeast (red), n-ary records regenerated from binary interaction records that are possibly represented n-ary data (green) and another set of regenerated n-ary records that are more conservative (red). There are 43% more yeast regenerated complexes from binary-represented complexes than yeast reported complexes (n-ary records) in iRefIndex. **b**. Distribution of complex sizes for n-ary records and regenerated complexes. Both distributions are skewed to the left, with the regenerated one being more strongly skewed. **c**. Distribution of complex sizes for n-ary records and a more conservative set of regenerated complexes. Records having more than 60 interactors are not shown in panels b and c.

iRefR includes an option to identify binary interactions that might be n-ary data represented as binary data (see option "include_generated_complexes" in function convert_MITAB_to_complexList). In these cases, a database may have chosen to represent n-ary data as a list of binary interactions using a spoke model representation. The list of binary interactions can be re-represented in a complexList. We term these "regenerated n-ary records". The set of interactions used to create a regenerated n-ary record is detected as a set of binary interactions that all 1) are curated by the same database from the same paper, 2) are supported by the same experimental method that is known to produce n-ary data (e.g. immunoprecipitation of a tagged protein from extract), and 3) share one protein (the hub of the spoke model), which corresponds to the experimental bait when this information is available. A total of 97072 yeast binary records meet these criteria and can be used to create 12046 regenerated n-ary records (Figure 4a). In contrast, there are only 6875 distinct canonical yeast n-ary records (three or more interactors in the record). This suggests that many n-ary data may be *disguised *as binary records. The number of protein interactors in yeast n-ary records ranges from 3 to 365 while regenerated n-ary records follow a different distribution (Kolmogorov-Smirnov test, p-value < 2.2e-16) that ranges in size from 3 to 1074 and is much more heavily left-distributed (Figure 4b). Many of the smaller regenerated n-ary records may be false - they may be derived from genuine independent binary observations (that just happen to all share one protein and the same method). Our method is unable to distinguish these cases and manual inspection of the original paper is required to confirm that n-ary data from one experiment is present. However, a more conservative selection of binary interactions can be used to regenerate n-ary records. Figure 4c shows the distribution of n-ary record sizes that were created using only binary records from BioGrid where MI:0004 (Affinity Chromatography) was listed as the interaction detection method. The same conclusions apply to this distribution (Kolmogorov-Smirnov test, p-value <2.2e-16). A number of methods can be used to detect binary interactions that are really representing n-ary data: most recently this has been carefully addressed in [22]. iRefIndex data and the iRefR package (see function "convert_MITAB_to_complexList") may be used to replicate or modify these methods by allowing the user to choose the experimental methods considered appropriate and allowing the user to either use only records with bait-prey information or groups of interactions sharing a common protein.

Arguably, it should not be up to analysts to *guess *which binary records are representing n-ary data and databases should adhere to common standards that clearly differentiate the two data types [22,23]. However, in the mean time, analysts should be aware of this potential problem. We show later, that the inclusion or exclusion of binary records that may be representing n-ary data can have a significant impact on node-degree distributions and, by proxy, network properties.

**Figure 5:**
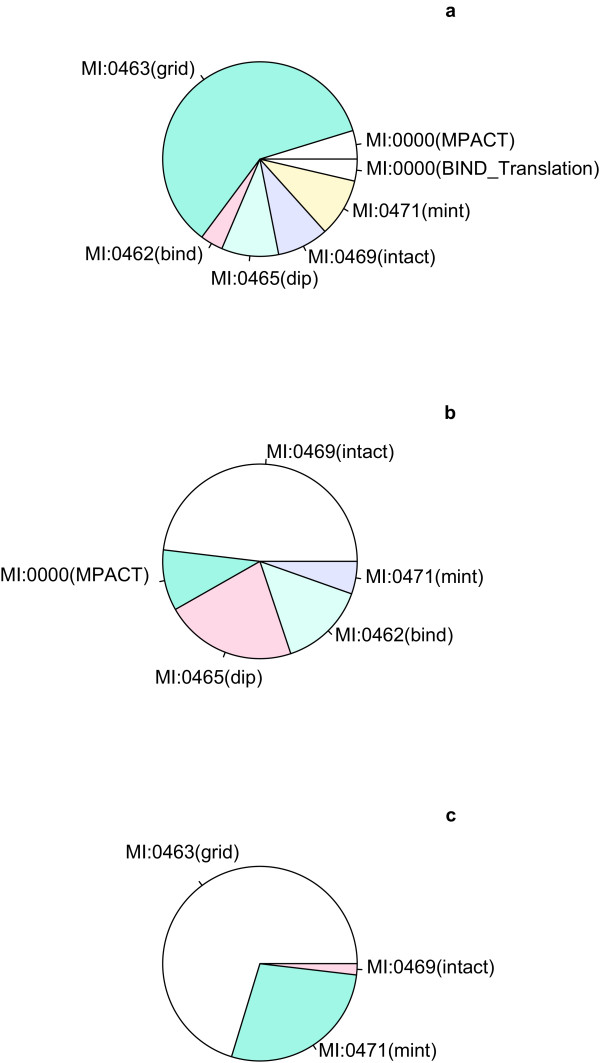
**Statistics per source database**.**a**. Division of all binary interaction records by source database. The majority of binary interactions come from the BioGrid database. The molecular interaction ontology controlled vocabulary term identifiers are given for each database (e.g. MI:0463 for BioGrid. **b**. Division of all n-ary interaction records (3 or more interactors per record) per source database. IntAct and DIP are the main sources. **c**. Binary interaction records used to create regenerated complexes per source database. The main source is BioGrid, followed by MINT. BioGrid represents all information as binary data (i.e. n-ary data is represented as binary data using a spoke-model).

Figure 5 and Table 4 show that the majority of regenerated n-ary records were derived from BioGrid while a smaller number were contributed by MINT and IntAct. This is consistent with BioGrid's curation policy to represent all data in a binary format. We are also aware that some high-throughput n-ary results are submitted by authors to IntAct as spoke-represented complexes (e.g. [24]) even though it is presently the policy of this database to represent n-ary data as lists of proteins and not lists of binary spoke interactions. Other examples are numerous and not limited to IntAct.

**Table 4:** Statistics for yeast regenerated complexes by source database.

database	binary interaction records	interaction records in generated complexes	generated complexes	low-throughput generated complexes
Biogrid	156860	35321	8971	6167
Mint	25358	14005	2715	1142
DIP	24810	0	0	0
IntAct	22351	932	360	267
MPACT	12395	0	0	0
BIND	9887	0	0	0

BioGrid contains 156860 yeast binary interaction records of which 35321 were used to create 8971 regenerated complexes (6167 of which were derived from low-throughput interactions). No spoke-represented complexes were detected in DIP, MPACT and BIND using the method described in the main text. The numbers in the last three columns are valid for the default list of experimental methods included in iRefR, which can be modified using the package.

### Selecting High or Low Throughput Data

Using iRefR, it is possible to select for a subset of interactions with a given bibliometric score. iRefIndex includes three such scores for each distinct interaction: (np, lpr, and hpr) [1]. The np score indicates the number of distinct publications (PubMed Identifiers) that support a given distinct interaction. The lpr score (lowest PubMed Identifier re-use) is the lowest number of distinct interactions that any publication (supporting this RIGID) is used to support. For example, a value of 1 indicates that at least one of the publications supporting this interaction has never been used to support any other interaction. This likely indicates that only one interaction was described by that reference and that the present interaction is not derived from high throughput methods. The hpr score (highest PubMed Identifier reuse) indicates the highest number of distinct interactions that any publication (supporting this interaction) is used to support.

**Figure 6:**
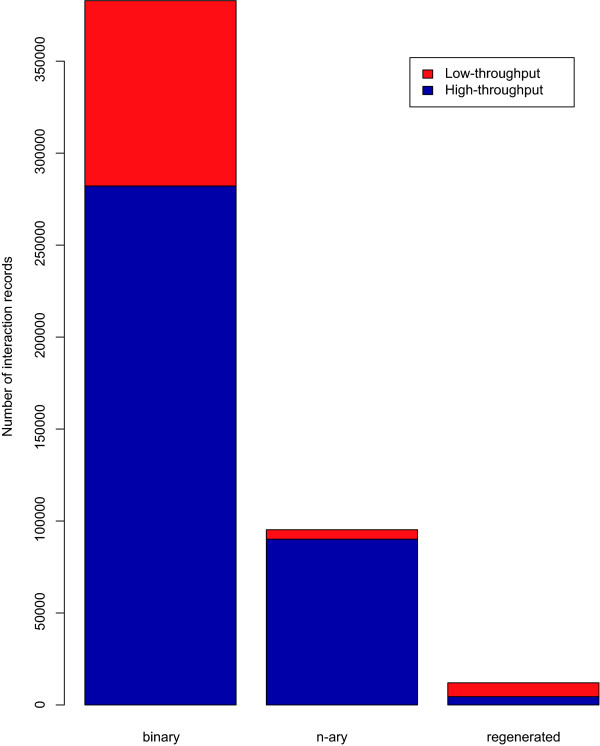
**High-throughput versus low-throughput yeast interaction data**. From a total number of 382903 yeast binary interaction records in iRefIndex, 26.3% are considered as low-throughput interactions (lpr < 22) versus 73.7% high-throughput records (lpr > 21). The set of yeast low-throughput n-ary data is only 5.4% of all 95263 n-ary interaction records. 62.9% of 12046 regenerated complex records (potential spoke-represented n-ary data) are derived from low-throughput binary interactions records.

As an example, two thirds of the regenerated complexes from BioGrid described in Table 4 are derived from binary interactions supported by low throughput experiments. In contrast, the overall breakdown of the yeast interactome data in Figure 6 shows that the majority of data is derived from high throughput studies. This indicates that the regenerated complexes from BioGrid may represent an important source of information about biological complexes that are derived from low-throughput experiments.

It is important to note that bibliometric scores alone (such as lpr) cannot be used as confidence scores to assess the reliability of an interaction [25,26]. Confidence scores may be generated using a number of methods and the user may wish to supplement their data with one or more of these depending on their requirements. The PSISCORE server acts as a meta-server to provide access to some of these methods and it can return confidence scores for interactions provided in MITAB format [27]. These scores are not included as part of the iRefIndex release.

### Graphical Representations

**Figure 7:**
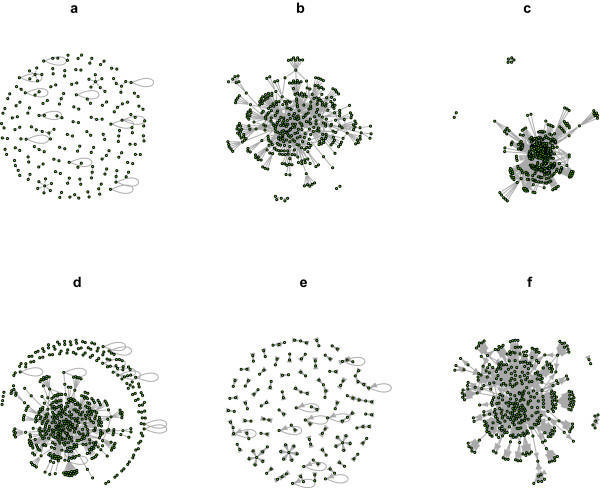
**Visual representation of interaction networks**. The figure presents six different subsets of the *E.coli *interactome from IntAct, generated by iRefR using the igraph package for R. **a**. binary data. **b**. n-ary data represented as a spoke model. **c**. n-ary data represented as a matrix model. **d**. binary and spoke-represented n-ary data. **e**. binary interactions with bait-prey information represented as a directed graph. **f**. spoke-represented n-ary data with bait-prey information represented as a directed graph.

**Figure 8:**
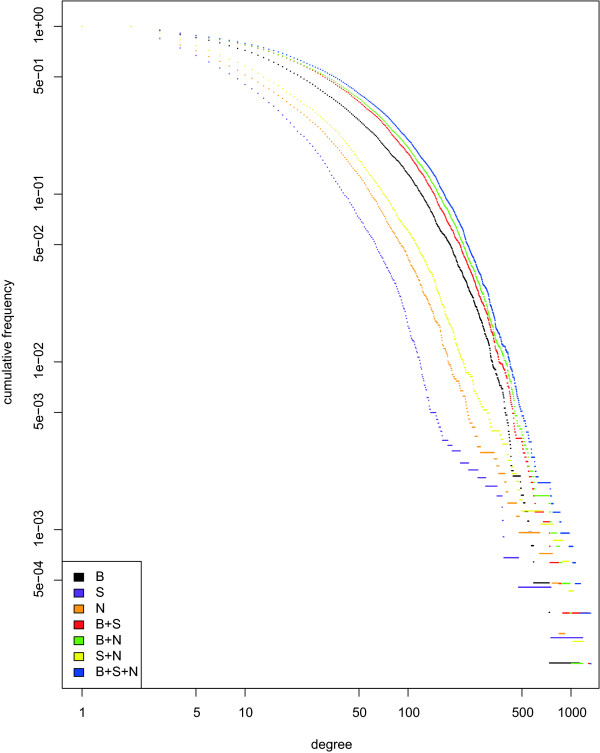
**Cumulative degree distributions for three data sets and their various combinations**. **B**: binary data. Yeast "true" binary interactions (binary interaction data minus data that is potentially n-ary data represented as binary data using a spoke-model: see S). **S**: spoke-represented n-ary data. Binary records that are potentially n-ary data represented as binary data using a spoke-model. **N**: n-ary data. Original interaction records that have three or more interactors. The figure corresponds to data in Table 6, showing potential power-law behaviour by some of these data sets.

The iRefR package allows the user to select interactomes and convert them to a format that can be read by three R graphical packages (*igraph*, *graph *and *RBGL *which in turn is dependent on the *graph *package). Figure 7 presents six different subsets of the *E.coli *interactome, generated by iRefR using the igraph package. Binary and n-ary interactions are shown, both in undirected and directed graphs, the latter ones showing available bait-prey information. Graphical studies can also be performed using iRefR data structures together with these other R packages. For example, Figure 8 shows cumulative node degree distributions for various subsets of yeast data from iRefIndex.

### Interactomes Comparison

Several different interactome data sets can be constructed using iRefR and their statistical properties assessed with R. In Table 5, a comparison was made between graphs for seven organisms in iRefIndex.

**Table 5:** Comparison of network properties for different model organisms.

a. C. elegans							
Dataset	n	I	X	C	dav	dvar	dmax
All canon spoke	5368	11848	11720	43	4.45 *	159.29	514
All non-canon spoke	5835	13774	13644	44	4.76	171.17	514
All canon matrix	5368	11848	11720	43	5.42*	210.28	514
LT canon spoke	570	544	498	41	2.59	26.13	92

**b. D. melanogaster**							
**Dataset**	**n**	**I**	**X**	**C**	**dav**	**dvar**	**dmax**

All canon spoke	9454	36576	36199	178	7.73	160.69	220
All non-canon spoke	11099	46727	46349	178	8.41	191.62	260
All canon matrix	9454	36576	36199	178	7.91	166.84	220
LT canon spoke	1496	3543	3371	153	4.91	53.90	101

**c. E. coli**							
**Dataset**	**n**	**I**	**X**	**C**	**dav**	**dvar**	**dmax**

All canon spoke	919	1065	924	109	2.10	0.98	13
All non-canon spoke	919	1065	924	109	2.10	0.98	13
All canon matrix	919	1065	924	109	2.14	1.17	13
LT canon spoke	786	826	783	17	2.08	0.83	11

**d. H. sapiens**							
**Dataset**	**n**	**I**	**X**	**C**	**dav**	**dvar**	**dmax**

All canon spoke	16320	107257	101792	5021	14.26	837.92	789
All non-canon spoke	18631	119764	114091	5226	13.92	816.91	789
All canon matrix	16320	107257	101792	5021	42.50	8637.40	1145
LT canon spoke	10860	44689	40655	3677	8.84	317.01	445

**e. M. musculus**							
**Dataset**	**n**	**I**	**X**	**C**	**dav**	**dvar**	**dmax**

All canon spoke	6280	9872	8501	1268	4.13*	144.41	608
All non-canon spoke	6560	10143	8741	1299	4.05	139.00	608
All canon matrix	6280	9872	8501	1268	83.50*	39515.35	1010
LT canon spoke	4811	5732	4777	865	3.36	112.71	608

**f. R. norvegicus**							
**Dataset**	**n**	**I**	**X**	**C**	**dav**	**dvar**	**dmax**

All canon spoke	2517	3212	2671	477	3.50	241.61	641
All non-canon spoke	2588	3304	2758	481	3.49	236.32	641
All canon matrix	2517	3212	2671	477	171.95	76175.86	858
LT canon spoke	1786	2263	1811	402	3.07	31.37	117

**g. S. cerevisiae**							
**Dataset**	**n**	**I**	**X**	**C**	**dav**	**dvar**	**dmax**

All canon spoke	6296	185384	178035	6875	65.43	8208.29	1322
All non-canon spoke	6299	185398	178045	6879	65.40	8206.43	1322
All canon matrix	6296	185384	178035	6875	157.76	51736.61	2292
LT canon spoke	4181	21981	21279	543	10.57	202.12	307

n: number of proteins

I: number of interaction records

X: number of binary interaction records

C: number of n-ary interaction records

dav: average protein degree

dvar: degree variance

dmax: maximum protein degree,

All: both HT and LT data

canon: canonical representation of proteins

spoke: spoke representation of complexes

matrix: matrix representation of complexes

Numbers are for the main seven organisms in iRefIndex

The two first rows only differ in their canonical representation and show that the number of nodes and interactions are reduced when proteins are considered in their canonical form but only for higher eukaryotes, where alternative splicing is present. However, this has very little effect on average degree, degree variance and maximum observed degree. Therefore, while canonicalization will affect search and retrieval of data, it does not impact greatly on average network properties.

In contrast, using the matrix representation for n-ary data can have a dramatic effect on average degree but this varies depending on the amount of n-ary data in the underlying data set; for instance, compare the change in average degree between rows 1 and 3 for *C. elegans *versus *M. Musculus *(* in Table 5). This highlights the importance of explicitly declaring which representation has been used to represent n-ary data.

We assessed properties for a low-throughput subset of the data (fourth row of each table). Here again the effects were organism specific (see underlined values in Table 5). The number of nodes in the *C. elegans *and *D. melanogaster *networks were reduced by 10-fold and 7-fold respectively if interaction records with an lpr score greater than 21 were removed (fourth row). However, the average degree fell less than two-fold. In contrast, the same low-throughput subset for human decreased the network size by 1.7 fold and the average degree by only 1.6 fold. Changes in network size and average degree were both less than 1.5-fold for mouse and rat. In contrast, the number of nodes in the low-throughput yeast interactome was also decreased in size by 1.5 fold but the average degree was decreased six-fold. These observations demonstrate that the same data filter can have vastly different effects on different interactomes.

### Degree distributions and power-law fitting

Table 6 summarizes an experiment with the degree distribution of the yeast network. A set of true Binary records (binary interactions after removal of possible spoke-represented n-ary data) has a degree distribution that does not follow the power-law model. We determined fit to a power-law using the algorithm described by Clauset *et al*. [28] where an exponent alpha is computed for the tail of the distribution where the degree is larger than a certain minimum value (xmin), and p-values less than 0.1 indicate that a power-law cannot be fitted to the data. P-values greater than 0.1 indicate a good fit (with the caveat that a different distribution might offer an even better fit).

**Table 6:** Effect of the inclusion of n-ary and/or regenerated complex data on the network degree distributions.

dataset	n	dmax	dmin	alpha	ntail	p_value	power-law
B	6227	1121	104	2.95	150	0.00	no
S	4406	1176	25	2.58	850	0.00	no
N	4164	912	96	3.32	189	0.32	yes
B + S	6281	1301	287	4.39	141	0.22	yes
B + N	6278	1180	273	4.23	183	0.08	no
S + N	4638	1192	137	3.48	159	0.50	yes
B + S + N	6294	1322	202	3.65	440	0.00	no

n: number of proteins in network

dmax: maximum node degree in network

dmin: minimum node degree considered for power-law fitting

alpha: fitted power-law exponent

ntail: number of nodes in tail of distribution considered for power-law fitting

p-value: power-law fitness- > 0.1 guarantees that the data can be fitted to a power-law model, however, better fits may exist for other functions

B: binary data

S: spoke-represented n-ary data

N: n-ary data

Likewise, the set of potential Spoke-represented n-ary records did not have support for a power-law distribution while the N-ary data could be fitted to a power law with a scaling coefficient of 3.3 (Table 6). The corresponding cumulative degree distributions are shown in Figure 8. The three data sets have visibly and statistically different node degree distributions.

Whether or not a combined data set can be fitted to a power-law is not intuitively obvious from its constituent data sets. For instance, Table 6 shows that the binary and spoke represented datasets together have moderate support for a power-law distribution while adding the n-ary data set removes this correspondence rather than enhancing it. Such combined networks and their inherent network properties are likely to have consequences for the analyses and methods that are so often applied to them in the literature. Just what this consequence is and whether it is significant is beyond the scope of this paper. However, we would submit that the three data sets could be considered different data types and speculate that any inferences drawn from their properties (or synthesized combinations) could just as likely be a consequence of how data are collected and represented as it is of any underlying biological reality.

In the very least, these results demonstrate the importance of specifying underlying data sets and data representation methods for n-ary data when calculating global network statistics.

A similar experiment in Table 7 shows that the yeast interactome variously does or does not potentially follow a power-law depending on the source database. Division of data by source database is an artificial one that we would not expect to alter network properties. These observations serve as a warning that choice of network simply by database may have unintentional effects.

**Table 7:** Comparison between the degree distribution properties of the yeast interactome by source database.

database	n	dmax	dmin	alpha	ntail	p-value	power-law
DIP	5189	351	79	3.46	177	0.04	no
BIND	5124	316	23	3.25	292	0.84	yes
IntAct	5893	969	55	3.03	439	0.26	yes
MINT	5523	390	33	3.33	310	0.92	yes
BioGrid	5794	1305	239	4.10	194	0.01	no
MPACT	4953	281	13	2.56	711	0.01	no
Total	6875	1327	199	3.63	459	0.00	no

The table shows the degree distribution properties of all seven databases reporting interactions with at least one yeast interactor in the record. The canonical representation is used to normalize protein interactors. N-ary data was represented using a spoke model. Data may include binary records that are representing n-ary data. Results may differ under other conditions.

Half of the databases (BIND, IntAct and MINT) suggest a power-law behaviour for the yeast interactome while the other half and the consolidated network cannot be fitted to a power-law.

n: number of distinct protein interactors in network

dmax: maximum node degree in network

dmin: minimum node degree considered for power-law fitting

alpha: fitted power-law exponent

ntail: number of nodes in tail of distribution considered for power-law fitting

p-value: power-law fitness- > 0.1 guarantees that the data can be fitted to a power-law model, however, better fits may exist for other functions

## Discussion

The iRefIndex addresses the need to consolidate protein interaction data into a single uniform data resource [1]. iRefR provides the user with access to this data source from an R environment.

We have demonstrated how data selection, redundancy resolution and n-ary data representation can affect network analysis. The iRefR package allows the user to specify how these issues are dealt with - this will facilitate communication of methods, reproducibility of network analyses and further modification and comparison of methods by researchers. We have emphasized the importance of explaining how an interactome has been constructed and how conclusions about interactome properties depend on this, and we have shown iRefR tools for selecting specific subsets of interest from different organisms, different databases, canonical or non-canonical representation of proteins, spoke or matrix model for n-ary data, subsets having bibliometric scores in a specified range (e.g. lpr), detection of potential binary-represented complexes, and data from specific experimental methods, source databases or publications, among others.

The iRefR package also eases many of the operations that are commonly carried out on this type of data. These include interconversion between MITAB, complexList, edgeList and graph formats, which simplify time-consuming data integration and file format manipulation procedures and, at the same time, allow the output to be exported to other applications and R packages.

The package includes additional features such as search and retrieval of data along with functions to deal with interconversion between ROGIDs and commonly used database accessions, as well as descriptive statistics. More advanced graphical statistics and operations can be performed using it together with the igraph or graph R packages, as explained in the software documentation.

The iRefIndex MITAB file is a major dependency of this package. In time, this dependency could be removed. The PSIMex Consortium [29] has recently introduced web-services that allow common querying and retrieval of interaction data in MITAB 2.5 format from several different interaction data providers (PSICQUIC web services [30]). At the time of writing, the MITAB format is in flux: PSICQUIC web services currently support MITAB 2.5 format while iRefIndex employs the latest 2.6 format. A 2.7 format is in the planning stage [31]. This effort aims to harmonize use of the MITAB format, n-ary data representation and introduce ROGID keys into all MITAB files provided by these services. Once this has been achieved, the iRefR package could be updated to make use of these services in a consistent and reliable manner.

## Conclusions

The iRefR package provides functionality to retrieve and work with data from the interaction Reference Index as well as to convert these data to formats useable by other graphical and statistical R-packages. The package addresses issues that are specific to working with this data type. We show that data selection, redundancy resolution and n-ary data representation all have an impact on graphical analysis. The package allows the user to control how these issues are dealt with and communicate them via an R-script written using the iRefR package.

## Methods

### R code

R code used to produce figures and tables in this paper are provided in Additional file 2. Power-law fitting code for R was kindly provided by Laurent Dubroca at http://tuvalu.santafe.edu/~aaronc/powerlaws/plfit.r according to methods described in [28].

### Version information

Analyses were carried out using version 8.0 of the iRefIndex [17]. The iRefR package version 0.93 is described in this paper and is compatible with version 7.0 and 8.0 of the iRefIndex. iRefR was constructed using version 2.13.1 of R [13].

### Availability

The iRefR package includes documentation for each function as well as a tutorial that guides the user through examples of each. The package is available from the Comprehensive R Archive Network http://cran.r-project.org/web/packages/iRefR/index.html and from ftp://ftp.no.embnet.org/irefindex/iRefR under a GPL license version 2 or higher http://www.gnu.org/licenses/gpl.html. Updates will be announced at http://irefindex.uio.no and its associated mailing list.

## Competing interests

The authors declare that they have no competing interests.

## Authors' contributions

AM and IMD designed the software. AM coded the R package. AM and IMD performed the analyses in the paper. AM and IMD wrote the paper. All authors read and approved the final manuscript.

## Supplementary Material

Additional file 1 **S1_iRefR_tutorial.pdf**. This tutorial guides the user through every function in the iRefR package. Click here for file

Additional file 2 **S2_R_Code_for_paper.txt**. R script showing code to generate data and figures for this paper. Click here for file
