# Supplementary material for: iRefR: an R package to manipulate the iRefIndex consolidated protein interaction database
Source: BMC Bioinformatics. 2011 Nov 24;12:455. doi: 10.1186/1471-2105-12-455 (PMC3282787; doi:10.1186/1471-2105-12-455)
Supplement: Additional file 1 — S1_iRefR_tutorial.pdf. This tutorial guides the user through every function in the iRefR package. [file 1471-2105-12-455-S1.PDF]

iRefR – An R package to manage the iRefIndex Consolidated Protein Interaction Database

Antonio Mora

Contents

1 Installing and Running **2**

2 Getting iRefIndex **2**

3 ID Conversion **5**
- 3.1 create_id_conversion_table 6
- 3.2 convert_protein_ID 6

4 Browsing the Database **7**
- 4.1 select_interaction_type 7
- 4.2 select_protein 8
- 4.3 select_database 10
- 4.4 select_confidence 11

5 Table format conversion **11**
- 5.1 convert_MITAB_to_edgeList 12
- 5.2 convert_edgeList_to_MITAB 13
- 5.3 convert_MITAB_to_complexList 14
- 5.4 convert_complexList_to_MITAB 15
- 5.5 merge_complexes_lists 16

6 Statistical functions **16**
- 6.1 summary_protein 16
- 6.2 summary_table 18

7 Graphical functions **19**
- 7.1 convert_edgeList_to_graph 20
- 7.2 convert_edgeList_to_graph -Alternative Exercise 22
- 7.3 convert_graph_to_edgeList 23
- 7.4 summary_graph 24

8 Bugs and Suggestions **25**

A Software needed to install packages from source using Windows **26**

B Code for selecting only human-human or E.coli-E.coli interactions **27**

iRefIndex is "an index of protein interactions available in a number of primary interaction databases including BIND, BioGRID, CORUM, DIP, HPRD, IntAct, MINT, Mpact, MPPI and OPHID... This index allows the user to search for a protein and retrieve a non-redundant list of interactors for that protein" (http://biotin.uio.no/wiki/iRefIndex).

iRefR is an R package that allows the user to load any version of the consolidated protein interaction database iRefIndex and perform tasks such as: selecting databases, publications, experimental methods, searching for specific proteins, separate binary interactions from complexes and polymers, convert different protein identifiers, generate complexes according to an algorithm that looks after possible misrepresented complexes, make general database statistics and create network graphs, among others.

The following tutorial introduces the basic use of this package. The details of each function and its arguments can be found in the package's help files, so you might want to use this tutorial and the help files together.

1 Installing and Running

To install the package from CRAN, type `install.packages("iRefR")`.

To install **iRefR** from source: If you are using Linux, copy the file `iRefR_0.93.tar.gz` to your `.../R/library/` directory. Open a terminal and run the gzipped file using `R CMD INSTALL iRefR_0.93.tar.gz -html -example`. If you are using Windows, a similar process can be followed, using the Windows built file (`R CMD INSTALL iRefR_0.93.zip -html -example`); however, R for Windows needs installing several tools in order to install packages from source. Some help to do that is provided in Appendix A.

iRefR depends on three packages: The **igraph** package, which can be installed from CRAN as explained before, and the packages **graph** and **RBGL**, which can be installed from `Bioconductor` as described in http://www.bioconductor.org/install/index.html.

Once installed, run R and call the package using:

```
> library(iRefR)
```

A warning message will appear indicating that the package **igraph** shares one function name with the package **graph** and one with **RBGL**. This is only relevant if you are planning to use those functions, in which case you must specify the package to use (f.ex., `igraph::degree`). If there are no error messages, you can start this tutorial. Read the headings of each exercise, run the examples in your R terminal and analyze the results. If you want to understand better the arguments and outputs of the functions, open the provided help file for the corresponding function (typing `?name-of-the-function`). This tutorial has been included in the folder `.../iRefR/doc`. The tutorial was tested using R 2.13.1.

2 Getting iRefIndex

get_irefindex allows the user to download the iRefIndex consolidated database from its FTP site. By default, the file(s) are copied to the working directory,

but you can also specify the path of a folder you can access, as explained in the documentation of the function.

```
> irefindex_80_mouse = get_irefindex("10090", "8.0", getwd())
```

```
Reading available iRefIndex file...
```

```
> irefindex_80_rat = get_irefindex(tax_id="10116", iref_version="8.0",
+  data_folder=getwd())
```

```
Reading available iRefIndex file...
```

```
> irefindex_80_cerevisiae = get_irefindex(tax_id="4932", data_folder=
+  getwd())
```

```
Reading available iRefIndex file...
```

```
> irefindex_80_celegans = get_irefindex(tax_id="6239")
```

```
Reading available iRefIndex file...
```

```
> irefindex_80_fly = get_irefindex("7227", "8.0")
```

```
Reading available iRefIndex file...
```

```
> irefindex_80_ecoli = get_irefindex(tax_id="562", iref_version="8.0")
```

```
Reading available iRefIndex file...
```

```
> irefindex_80_human = get_irefindex("9606", "8.0")
```

```
Reading available iRefIndex file...
```

```
> irefindex_curr_human = get_irefindex("9606", "current", getwd())
```

```
Reading available iRefIndex file...
```

```
> irefindex_curr_ecoli = get_irefindex("562", data_folder=getwd())
```

```
Reading available iRefIndex file...
```

Note: To june 2011, current="8.0".

Edit one of the tables to see the contents of an iRefIndex flat file. You will see that every line corresponds to an interaction between a pair of proteins, and every column stores diferent types of information about that interaction. This format is called MITAB 2.6 (see: http://irefindex.uio.no/wiki/README_MITAB2.6_for_iRefIndex_7.0).

```
edit(irefindex_curr_human[1:10,])
```

The Human iRefIndex version 8.0 contains 355104 interaction entries with 54 different types of information per interaction. Read the column names to find out what type of information is available.

```
> dim(irefindex_curr_human)
```

```
[1] 355104     54
```

```
> colnames(irefindex_curr_human)
```

```
 [1] "X.uidA"                 "uidB"
 [3] "altA"                   "altB"
 [5] "aliasA"                 "aliasB"
 [7] "method"                 "author"
 [9] "pmids"                  "taxa"
[11] "taxb"                   "interactionType"
[13] "sourcedb"               "interactionIdentifier"
[15] "confidence"             "expansion"
[17] "biological_role_A"      "biological_role_B"
[19] "experimental_role_A"    "experimental_role_B"
[21] "interactor_type_A"      "interactor_type_B"
[23] "xrefs_A"                "xrefs_B"
[25] "xrefs_Interaction"      "Annotations_A"
[27] "Annotations_B"          "Annotations_Interaction"
[29] "Host_organism_taxid"    "parameters_Interaction"
[31] "Creation_date"          "Update_date"
[33] "Checksum_A"             "Checksum_B"
[35] "Checksum_Interaction"   "Negative"
[37] "OriginalReferenceA"     "OriginalReferenceB"
[39] "FinalReferenceA"        "FinalReferenceB"
[41] "MappingScoreA"          "MappingScoreB"
[43] "irogida"                "irogidb"
[45] "irigid"                 "crogida"
[47] "crogidb"                "crigid"
[49] "icrogida"               "icrogidb"
[51] "icrigid"                "imex_id"
[53] "edgetype"               "numParticipants"
```

However, those entries correspond to only 138570 distinct interactions, due to the fact that some interactions are reported more than once, either for a different source database, experimental method, etc. Each distinct interaction has an identifier called `rigID`, which is the one we have used to retrieve this information.

```
> length(unique(irefindex_curr_human$irigid))
```

```
[1] 138570
```

Compare the known number of protein interactions for all available organisms in `iRefIndex`:

```
> list(ecoli=dim(irefindex_80_ecoli)[1],
+  scerevisiae=dim(irefindex_80_cerevisiae)[1],
+  celegans=dim(irefindex_80_celegans)[1],
+  fly=dim(irefindex_80_fly)[1],
+  mouse=dim(irefindex_80_mouse)[1], rat=dim(irefindex_80_rat)[1],
+  human=dim(irefindex_80_human)[1])
```

```
$ecoli
[1] 16058

$scerevisiae
[1] 480908

$celegans
[1] 41848

$fly
[1] 154229

$mouse
[1] 49064

$rat
[1] 20309

$human
[1] 355104
```

It is important to highlight that `iRefIndex` calls "human interactions" not only to the interactions between two human proteins but also to any interaction between a human protein and a protein from a different organism (for example, a virus or a bacterium). This tutorial will work with all these interactions. In case the user is interested only in human-human interactions, there is a simple piece of code provided in Appendix B that could be run at this point.

3 ID Conversion

`iRefIndex` guarantees the non-redundancy of protein information by assigning a different protein identifier (called Redundant Object Group, ROG) to every different protein sequence. This is called the `non-canonical` representation of a protein. At the same time, groups of non-redundant proteins might be different isoforms of a given protein, and, in this case, the identifier of one protein of the group (called cROG or canonical ROG) is chosen to represent the entire group of similar proteins. This is called the `canonical` representation of proteins. The iROG and icROG identifiers correspond to integer representations of the ROG and cROG, respectively.

Most of the **iRefR** functions work with iROGs and icROGs, but the user might be interested in convert these IDs to a more traditional protein ID, or, in general, convert between two types of protein IDs. There are two main functions in **iRefR** to handle protein ID conversion: **create_id_conversion_table** allows to generate a table to convert identifiers between the most important protein identifiers; and **convert_protein_ID** performs the actual conversion from an ID type to another ID type.

3.1 create_id_conversion_table

```
> id_conversion_table_ecoli =
+  create_id_conversion_table(irefindex_80_ecoli, "data",
+  "id_conversion_table_562_a")
```

```
Generating ID Conversion table...
25% completed...
50% completed...
75% completed...
Conversion table has been copied to:
/div/dias/u2/antonimo/R-2.13.1/library/iRefR/data/id_conversion_table_562_a.txt
```

```
> id_conversion_table_human =
+  create_id_conversion_table(irefindex_80_human, "data",
+  "id_conversion_table_9606_a")
```

```
Generating ID Conversion table...
25% completed...
50% completed...
75% completed...
Conversion table has been copied to:
/div/dias/u2/antonimo/R-2.13.1/library/iRefR/data/id_conversion_table_9606_a.txt
```

```
edit(id_conversion_table_human[1:10,])
```

Note: Approx. Time (default ID types): E.coli: 4 s; H.sapiens: 3'; All organisms: 18'

This table only needs to be generated once, after getting `iRefIndex`, and does not need to be manipulated. When doing an actual ID conversion, the table will be open by **iRefR** using the next function.

Only proteins that have known reported protein-protein interactions are included in this table. If a protein ID has no reported interactions, ID conversion is not possible, given that such a protein wouldn't be relevant for the present package.

3.2 convert_protein_ID

```
> ## converting from geneID to uniprotID
> uniprot_id_value = convert_protein_ID("entrezgene/locuslink", "23214",
+  "uniprotkb", id_conversion_table_human)
```

The output are two values (`A1L3W4` and `Q96QU8`), indicating that the gene ID 23214 corresponds to two different UniProt IDs.

```
> ## converting a list of iROG IDs to RefSeq IDs
> irog_list = c("4772972", "3303645", "3196230", "10724600", "3254823",
+  "3130413")
> refseq_list = list()
> for (i in irog_list) {
+         refseq_list[[i]] = convert_protein_ID("irogid", i, "refseq",
+  id_conversion_table_human)
+ }
```

The output is an R list, where each list element is named after one of the iROGs in the original list, and the content of such a list element might be either one value or a vector of values, depending on the case that a given iROG represents either one or more RefSeq IDs.

4 Browsing the Database

`iRefIndex` stores information of several databases, proteins and three types of interactions (binary, complex and polymer). The following functions are useful to get the specific subset of `iRefIndex` according to the needs of the user:

select_interaction_type, **select_protein**, **select_database** and **select_confidence** select the records belonging to a specified interaction type, protein ID, primary database or confidence score, respectively.

4.1 select_interaction_type

```
> iRef_binary = select_interaction_type("binary", irefindex_curr_human)
> iRef_complex = select_interaction_type("complex", irefindex_curr_human)
> iRef_polymer = select_interaction_type("polymer", irefindex_curr_human)
```

Generate a pie with the number of binary, complex and polymer records in `iRefIndex`. Roughly 7/8 of the human interaction records are binary interactions, while roughly 1/8 corresponds to complex interactions.

```
> pie_int_type = pie(c(dim(iRef_binary)[1], dim(iRef_polymer)[1],
+  dim(iRef_complex)[1]),labels=c("Binaries","Polymers","Complexes"),
+  main="iRefIndex Records per Interaction Type")
```

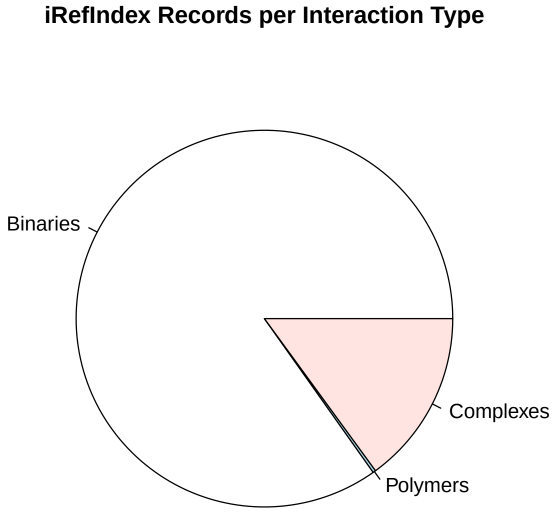

Take a look at the tables of binaries and complexes and observe the differences between the `interactionType` and `numParticipants` columns. You can see that X is the tag for binary interactions while C is the tag for complexes. Binary interactions have always 2 participants, while complexes have more than two.

```
edit(iRef_binary[1:5,])
edit(iRef_complex[1:5,])
```

We can also check the number of records and the number of distinct interactions per interaction type: There are 371244 reported binary interaction records, corresponding to 132889 distinct binary interactions, while there are 122486 complex records, corresponding to 5232 complexes.

```
> number_binary_interactions = dim(iRef_binary)[1]
> number_distinct_binary_interactions =
+  length(unique(iRef_binary$irigid))
> number_complex_interactions = dim(iRef_complex)[1]
> number_distinct_complex_interactions =
+  length(unique(iRef_complex$irigid))
> list(number_binary_interactions = number_binary_interactions,
+  number_distinct_binary_interactions =
+  number_distinct_binary_interactions,
+  number_complex_interactions = number_complex_interactions,
+  number_distinct_complex_interactions =
+  number_distinct_complex_interactions)

$number_binary_interactions
[1] 301129

$number_distinct_binary_interactions
[1] 132889

$number_complex_interactions
[1] 53125

$number_distinct_complex_interactions
[1] 5232
```

4.2 select_protein

```
> output_1 = select_protein("irogid", "4566610", irefindex_80_human,
+  "not_full_complex")        #P53
> output_2 = select_protein("icrogid", "4566610", irefindex_80_human,
+  "not_full_complex")
> output_3 = select_protein("icrogid", "3484827", irefindex_80_human,
+  "not_full_complex")
> output_4 = select_protein("icrogid", "3484827", irefindex_80_human,
+  "full_complex")
```

Note that icROG stands for the iRefIndex integer identifier of the canonical representation of the protein, while iROG stands for the non-canonical version

(for the distinction between canonical and non-canonical proteins please read above or go to the documentation of this function). The `full_complex` option indicates that we have retrieved all the interaction records presented in every complex, besides the ones containing that protein, while `not_full_complex` limits the report to only the lines containing that protein, as shown in the documentation of the function.

In the previous exercise, we have recovered all interaction records for one protein (irogid=4566610) and its canonical counterpart (irogid=3484827). In the first case, the search retrieves 46 records in iRefIndex/MITAB format. The second case does not retrieve any record, meaning that such a protein identifier does not exist. This is because the canonical protein representing the irogid=4566610 is not 4566610 but 3484827, and 4566610 was never used as a canonical protein. We can know that by converting the irogid to icrogid.

```
> dim(output_1)

[1] 46 54

> dim(output_2)

[1]  0 54

> convert_protein_ID("irogid", "4566610", "icrogid",
+  id_conversion_table_human)

[1] "3484827"
```

In the third exercise, we found that there are 236 records for the canonical protein of that canonical group. We can find all the irogids for such a group, to discover that this canonical group contains 68 different proteins, including 3484827 and 4566610. The fourth case is the same third case but adding the `full_complex` parameter. We can observe the number of records going from 236 to 378 for this reason.

```
> dim(output_3)

[1] 236  54

> unique(c(output_3$irogida, output_3$irogidb))

 [1]  3484827  5831940  4566610  4831970  2186087  1505377  2697990  4724590
 [9] 16583742  4497269  5365786  1961605  4303895  2428575  5824296  4972844
[17]  4497883  4122724  4435463   746078  4691983  4629164  4041918  4577585
[25]  2886402  4867693  2413104 13976011  5335424  1208032  1885413  2857551
[33]  5310451 14005586   517184 16751042 13987717  4615198   878471  5017653
[41]  4819293  4826045  2791218   991986  5254792  4728973  1636625   793231
[49]  5821280  3995154  2583546 11793640  4835255   660830 16743445 16743450
[57] 16743446 16743454   548553 16743443 16743449 16743453 16743452 16743447
[65] 16743442 16743444 16743451 16743448

> dim(output_4)

[1] 378  54
```

iRefR also allows the user to perform a selection of multiple proteins at the same time. Two examples of this are shown below. In the first case, a list of tables is generated using a simple `for` loop. In the second case, the vector of protein identifiers is directly introduced in the function, generating a single iRefIndex MITAB table as an output. The first one might be faster while the second one allows the user to use the output directly into any other **iRefR** function.

```
> irog_list = c("4772972", "3303645", "3196230", "10724600", "3254823",
+  "3130413")
> table_list = list()
> for (i in irog_list) {
+         table_list[[i]] = select_protein("irogid", i,
+  irefindex_80_human, "not_full_complex")
+ }
> table_single = select_protein("irogid", irog_list, irefindex_80_human,
+  "not_full_complex")
```

4.3 select_database

```
> binary_INTACT = select_database("intact", iRef_binary,
+  "this_database")
> binary_non_INTACT = select_database("intact", iRef_binary,
+  "except_this_database")
> complex_INTACT_CORUM = select_database(c("intact", "CORUM"),
+  iRef_complex, "this_database")
> complex_non_INTACT_CORUM = select_database(c("intact", "CORUM"),
+  iRef_complex, "except_this_database")
```

In the first exercise, only the binary records coming from the primary database `IntAct` has been selected. In the second case, all binary records of `iRefIndex` are selected except for those coming from `IntAct`. The third case selects all complex records coming from the primary databases `IntAct` and `CORUM`. The last case, all complexes records after removal of `IntAct` and `CORUM`.

```
> dim(binary_INTACT)

[1] 39969    54

> dim(binary_non_INTACT)

[1] 261160     54

> dim(complex_INTACT_CORUM)

[1] 26313    54

> dim(complex_non_INTACT_CORUM)

[1] 26812    54
```

By default, `iRefIndex` includes both experimental and computationally predicted interactions. Besides allowing the user to make analyses per database, this function can be also important to include or exclude predicted interactions in the analysis, which can be done by selecting the `OPHID` database (select only predicted), or excluding it (excluding predicted).

4.4 select_confidence

```
> high_confidence_complexes = select_confidence("lpr", c(1, 3:10),
+  iRef_complex)
> dim(high_confidence_complexes)
```

```
[1] 19434    54
```

`iRefIndex` includes three bibliometric confidence scores of an interaction: The lpr score (lowest pmid re-use) is the lowest number of distinct interactions (irigids) that any PMID (supporting the interaction in this row) is used to support. A value of 1 indicates that at least one of the PMIDs supporting this interaction has never been used to support any other interaction. This likely indicates that only one interaction was described by that reference and that the present interaction is not derived from high throughput methods. Other scores are defined in the documentation and the iRefIndex website.

In the exercise, we have asked for the set of complex interactions whose lpr-score is equal to 1 or between 3 and 10. The result is a subset of 19434 complex interaction records.

5 Table format conversion

There are three types of table formats in **iRefR**: MITAB format, complexList format and edgeList format.

The MITAB format is an international standard where each line represents either an interaction between two proteins or between the same protein or the membership of a protein to a complex, and it is described here: `http://biotin.uio.no/wiki/README_iRefIndex_MITAB_7.0`. The iRefIndex MITAB format includes 54 columns with different types of information regarding each interaction.

The complexList format is a table with two columns, where the first column corresponds to a group identifier and the second column to a group of proteins, commonly a complex, written as a comma-separated string. This representation has less information than the MITAB but simplifies the visualization of groups of proteins, such as complexes. A similar format is used by complex databases such as `CORUM`.

Finally, the edgeList format offers a list of edges and their weights, coming from binary interactions and/or complexes that has been represented using a spoke, bipartite or matrix model. This format is mainly used for graphical purposes.

There are five functions related to table format conversion:
convert_MITAB_to_edgeList,
convert_edgeList_to_MITAB,
convert_MITAB_to_complexList and
convert_complexList_to_MITAB do the format conversions described in their names. **merge_complexes_lists** merges an ordered list of tables in complexList format.

5.1 convert_MITAB_to_edgeList

```
> ## undirected edges (to generate undirected graphs):
> all_INTACT = select_database("intact", irefindex_curr_ecoli,
+  "this_database")
> binary_INTACT = select_interaction_type("binary", all_INTACT)
> complex_INTACT = select_interaction_type("complex", all_INTACT)
> edgeList_binary_INTACT = convert_MITAB_to_edgeList(binary_INTACT)
> edgeList_complex_INTACT_s = convert_MITAB_to_edgeList(
+  complex_INTACT, "default", "spoke")
> edgeList_complex_INTACT_m = convert_MITAB_to_edgeList(
+  complex_INTACT, "default", "matrix")
> edgeList_all_INTACT = convert_MITAB_to_edgeList(all_INTACT,
+  "default", "spoke")
```

We have generated four edgeLists: a binary set, a spoke-represented complex set, a matrix-represented complex set and the whole database using spoke-represented complexes. Note that the matrix model is more time-consuming. Generating an edgeList for the whole human `iRefIndex` using a matrix model can take less than 2 minutes. As expected, spoke models generate less edges than matrix models (834 vs 5636).

```
> dim(edgeList_binary_INTACT)

[1] 123   3

> dim(edgeList_complex_INTACT_s)

[1] 834   3

> dim(edgeList_complex_INTACT_m)

[1] 5636    3

> dim(edgeList_all_INTACT)

[1] 951   3
```

```
edit(edgeList_binary_INTACT[1:5,])
edit(edgeList_complex_INTACT_s[1:5,])
```

```
> ## directed edges (to generate directed bait-prey graphs):
> edgeList_binary_INTACT_dir = convert_MITAB_to_edgeList(
+  binary_INTACT, "default", "bipartite", "yes", "directed")

[1] "Only rows with bait information are included in directed edgeLists"

> edgeList_complex_INTACT_sdir = convert_MITAB_to_edgeList(
+  complex_INTACT, "default", "spoke", "yes", "directed")
> edgeList_all_INTACT_dir = convert_MITAB_to_edgeList(all_INTACT,
+  "default", "spoke", "yes", "directed")

[1] "Only rows with bait information are included in directed edgeLists"
```

Here we generated three directed examples. In a directed edgeList, the arrow will point from the first node to the second node. Compare the undirected edgeList to the directed one: Only 108 binary edges (versus 123 in the undirected case) while 849 complex-spoke edges (versus 834 in the undirected case). The reason for having less binary edges is that only rows with bait information are included in binary directed edgeLists. The reason of having more complex-spoke edges is that some complexes have more than one bait, giving origin to bidirectional edges (represented in an edgeList as two different edges in opposite direction).

```
> dim(edgeList_binary_INTACT_dir)
```

```
[1] 108   3
```

```
> dim(edgeList_complex_INTACT_sdir)
```

```
[1] 849   3
```

```
> dim(edgeList_all_INTACT_dir)
```

```
[1] 954   3
```

5.2 convert_edgeList_to_MITAB

```
> new_binary = convert_edgeList_to_MITAB(edgeList_binary_INTACT,
+  irefindex_curr_ecoli, "yes", "binary")
> reconstructed_binary_INTACT = select_database("intact",
+  new_binary, "this_database")
> setequal(dim(binary_INTACT), dim(reconstructed_binary_INTACT))
```

```
[1] TRUE
```

```
> new_complex_s = convert_edgeList_to_MITAB(
+  edgeList_complex_INTACT_s, irefindex_curr_ecoli, "yes",
+  "complex")
> reconstructed_complex_INTACT_s = select_database("intact",
+  new_complex_s, "this_database")
> setequal(dim(complex_INTACT), dim(reconstructed_complex_INTACT_s))
```

```
[1] TRUE
```

```
> new_all = convert_edgeList_to_MITAB(edgeList_all_INTACT,
+  irefindex_curr_ecoli)
> reconstructed_all_INTACT = select_database("intact", new_all,
+  "this_database")
> setequal(dim(all_INTACT), dim(reconstructed_all_INTACT))
```

```
[1] TRUE
```

In this exercise, we have reconstructed the original MITAB tables from the edgeLists we generated in the previous exercise. The human case is more time-consuming and the cases with the whole database and the matrix model can take several hours. Note that the dimensions would not be equal for the directed case at previous examples because only records with baits can be reconstructed. Directed edgeLists only include information with baits, so that all other cases are ignored during edgeList generation and there is no way to go back to the complete original MITAB.

5.3 convert_MITAB_to_complexList

```
> known_complexes_ecoli_complexList =
+  convert_MITAB_to_complexList(irefindex_curr_ecoli,
+  canonical_rep="yes", include_generated_complexes="no",
+  list_methods="default")
> generated_complexes_ecoli_complexList =
+  convert_MITAB_to_complexList(
+  select_interaction_type("binary", irefindex_curr_ecoli),
+  canonical_rep="yes", include_generated_complexes="yes",
+  list_methods="default")
> all_complexes_ecoli_complexList = convert_MITAB_to_complexList(
+  irefindex_curr_ecoli, canonical_rep="yes",
+  include_generated_complexes="yes", list_methods="default")
```

This function does convert a table from MITAB format to complexList format. To do that, it does convert all interactions listed as complexes in the MITAB table and, optionally, check if some groups of binary interactions can be interpreted as complexes (possibly misrepresented or binary-represented), according to an **iRefR** algorithm that searches for groups of proteins coming from co-precipitation experiments, sharing the same PMID, source database and bait. In this exercise, we generate three complexLists: one for the known complexes (124 complexes), one for the generated complexes from binary data (505 complexes) and one for the combined case. Note that the names of the known complexes are just the irigids or icrigids, while the names of generated complexes are composed of the source database, PMID, experimental method and bait they were generated from.

```
> dim(known_complexes_ecoli_complexList)

[1] 124   2

> dim(generated_complexes_ecoli_complexList)

[1] 505   2

> dim(all_complexes_ecoli_complexList)

[1] 629   2

edit(known_complexes_ecoli_complexList[1:5,])
edit(generated_complexes_ecoli_complexList[1:5,])
```

This function might generate repeated complexes; f.ex. when the same complex is generated from two different sources. If the user wants a list of unique complexes, the `unique` function must be applied after generating the complexList. In this case, 628 unique complexes.

```
> length(unique(all_complexes_ecoli_complexList[,2]))

[1] 628
```

5.4 convert_complexList_to_MITAB

```
> reconstructed_known_ecoli_MITAB =
+  convert_complexList_to_MITAB(known_complexes_ecoli_complexList,
+  irefindex_curr_ecoli, "yes", "no")
> reconstructed_generated_ecoli_MITAB =
+  convert_complexList_to_MITAB(
+  generated_complexes_ecoli_complexList, irefindex_curr_ecoli,
+  "yes", "yes")
> reconstructed_all_ecoli_MITAB = convert_complexList_to_MITAB(
+  all_complexes_ecoli_complexList, irefindex_curr_ecoli,
+  "yes", "yes")
```

In this exercise, we have reconstructed the original MITAB tables from the complexLists we generated in the previous exercise. Note that, in the first case (only real known complexes), all complex interactions were correctly reconstructed. However, in the second and third cases, smaller tables are reconstructed: That is because only the known complexes plus the binary interactions that were used to construct complexes can be recovered; the other binaries were previously ignored.

```
> setequal(dim(select_interaction_type("complex", irefindex_curr_ecoli)),
+  dim(reconstructed_known_ecoli_MITAB))

[1] TRUE

> setequal(dim(select_interaction_type("binary", irefindex_curr_ecoli)),
+  dim(reconstructed_generated_ecoli_MITAB))

[1] FALSE

> setequal(dim(irefindex_curr_ecoli), dim(reconstructed_all_ecoli_MITAB))

[1] FALSE
```

It is possible, however, to use the reconstructed MITAB tables to regenerate the complexList tables and compare them to the originals. In this case, it is reported that the functions are successful converting forth and back between these two formats.

```
> reconstructed_generated_complexList =
+  convert_MITAB_to_complexList(
+  reconstructed_generated_ecoli_MITAB, canonical_rep="yes",
+  include_generated_complexes="yes")
> setequal(dim(generated_complexes_ecoli_complexList),
+  dim(reconstructed_generated_complexList))

[1] TRUE

> reconstructed_all_complexList = convert_MITAB_to_complexList(
+  reconstructed_all_ecoli_MITAB, canonical_rep="yes",
+  include_generated_complexes="yes")
> setequal(dim(all_complexes_ecoli_complexList),
+  dim(reconstructed_all_complexList))

[1] TRUE
```

5.5 merge_complexes_lists

```
> consolidated_complexList = merge_complexes_lists(
+ list(known_complexes_ecoli_complexList,
+  generated_complexes_ecoli_complexList))
```

The new table will merge the two previous ones, but repeated complexes will stay only in the first (known complexes) table. One application of this function might be finding the number of repeated complexes both inside each of the lists and between the two lists. In this case, 1 complex has been repeated.

```
> print(paste(
+  "Number_complexes_repeated_inside_lists_or_between_lists = ",
+  dim(known_complexes_ecoli_complexList)[1] + dim(
+  generated_complexes_ecoli_complexList)[1] - dim(
+  consolidated_complexList)[1]))

[1] "Number_complexes_repeated_inside_lists_or_between_lists =  1"
```

6 Statistical functions

iRefR contains two main functions to easily report statistics: **summary_protein** gives the user information on interactors and complexes, for a given protein; and **summary_table** gives statistical information about some of the columns of a given iRefIndex/MITAB table.

6.1 summary_protein

```
> output_1 = summary_protein("irogid", "4566610", irefindex_80_human)
> output_2 = summary_protein("icrogid", "4566610", irefindex_80_human)
> output_3 = summary_protein("icrogid", "3484827", irefindex_80_human)
```

Using the same example than in the **search_protein** function (p53 protein), we have made summaries of the proteins (instead of recovering the MITAB table). In the first example, the non-canonical protein 4566610 presents 14 binary interactions (14 distinct interactors), 1 complex (with 4 additional subunits) and no polymer interactions. The second example is the search for a non-existent canonical ID and the third one is the search with the right canonical ID, giving us 88 binary interactions and 3 complexes, corresponding to all the proteins of the canonical group.

```
> output_1

$summary_interactions_per_protein
                       table_names table_values
1                           irogid      4566610
2 number_interactions_X(#irigids)           14
3 number_interactions_C(#irigids)            1
4 number_interactions_Y(#irigids)            0
5            total_degree(#irigs)           15
```

```
$interactors_binary
 [1] 1208032 1885413 2413104 2428575 2583546 2857551 2886402 3995154 4041918
[10] 4629164 4724590 4831970 4835255 5335424

$interactors_complex
[1] "2399757,2447260,4041918,5335424"

$interactors_polymer
integer(0)

> output_2

$summary_interactions_per_protein
[1] "ERROR. Protein ID not found."

$interactors_binary
[1] "ERROR. Protein ID not found."

$interactors_complex
[1] "ERROR. Protein ID not found."

$interactors_polymer
[1] "ERROR. Protein ID not found."

> output_3

$summary_interactions_per_protein
                      table_names table_values
1                         icrogid      3484827
2 number_interactions_X(#icrigids)           88
3 number_interactions_C(#icrigids)            3
4 number_interactions_Y(#icrigids)            0
5            total_degree(#icrigs)           91

$interactors_binary
 [1]   276591   517184   548553   746078   878471   991986  1208032  1505377
 [9]  1636625  1885413  1961605  2186087  2428575  2583546  2697990  2791218
[17]  2857551  2886402  3995154  4041918  4122724  4303895  4435463  4497269
[25]  4497883  4577585  4629164  4691983  4724590  4728973  4819293  4826045
[33]  4831970  4835255  4867693  4972844  5017653  5254792  5310451  5335424
[41]  5365786 11793640 14005586 16743442 16743443 16743444 16743445 16743446
[49] 16743447 16743448 16743449 16743450 16743451 16743452 16743453 16743454

$interactors_complex
[1] "2399757,2447260,4041918,5335424"
[2] "66692,201818,321631,351104,369866,390328,399772,448835,746078,862702,899337,931820,10
[3] "2583,29715,50293,105791,327364,632859,775439,804277,972989,1017180,1309159,1471920,14

$interactors_polymer
integer(0)
```

6.2 summary_table

```
> general_human_statistics = summary_table(irefindex_80_human)
> biogrid_statistics = summary_table(select_database("grid",
+  irefindex_80_human, "this_database"))
> complexes_statistics = summary_table(select_interaction_type("complex",
+  irefindex_80_human))
> zhu_paper_statistics = summary_table(
+ irefindex_80_human[which(irefindex_80_human$pmids == "pubmed:9989503"),])
> low_thruput_statistics = summary_table(select_confidence("lpr", c(1,2),
+  irefindex_80_human))
> p53_statistics = summary_table(select_protein("irogid", "4566610",
+  irefindex_80_human, "not_full_complex"))
```

This function allows the user to see a summary of a MITAB table containing the number of non-canonical interactions, canonical interactions, non-canonical proteins, canonical proteins, publications and experimental methods, as well as the distribution of records per source database, records per interaction type and records per number of participants in the interactions. The first example contains the general statistics for the whole human dataset. The second example is restricted to the BioGrid database: note that all interactions are binary, and the reason of this is that BioGrid stores complexes directly in a spoke-represented manner. The third example does the same for all complexes, the fourth one for one specific paper (note that these interactions have been reported by several databases), the fifth one for all interactions with an lpr of 1 or 2 (assumed to be low throughput) and the sixth one for one specific protein.

```
> general_human_statistics

$number_non_canonical_interactions
[1] 138570

$number_canonical_interactions
[1] 125898

$number_non_canonical_proteins
[1] 33180

$number_canonical_proteins
[1] 30690

$number_publications
[1] 26909

$number_experimental_methods
[1] 1632

$source_db_distribution

MI:0000(BIND_Translation)            MI:0000(CORUM)             MI:0000(MPACT)
                     2674                      8666                         12
```

```
           MI:0000(MPPI)           MI:0000(ophid)           MI:0462(bind)
                     828                    58427                   26521
           MI:0463(grid)             MI:0465(dip)           MI:0468(HPRD)
                   52423                    25190                   93154
         MI:0469(intact)            MI:0471(mint)
                   58398                    28811

$interaction_type_distribution

     C      X      Y
 53125 301129    850

$number_participants_distribution

     1      2      3      4      5      6      7      8      9     10     11
   850 301129  11147   5453   3789   2290   1778   1570   1230   1301   1076
    12     13     14     15     16     17     18     19     20     21     22
  1185   1164    756    773    699    696    657    581    613    420    677
    23     24     25     26     27     28     29     30     31     32     33
   408    339    376    337    458    551    549    496    246    320    491
    34     35     36     37     38     39     40     41     42     43     44
   302    318    143    246    114     39    130    164    293    129     88
    45     46     47     48     49     51     52     53     54     55     56
    45    366    235    143     49     51     52    106    108    110    112
    58     59     60     61     62     63     64     65     67     68     69
   114    155     61    122     62    188    190    194    200    134     68
    70     71     73     75     77     78     79     80     81     82     85
   208    141     72    297    154     78    156    159     81    243    169
    86     87     88     90     95    100    101    102    104    107    110
    82     87    176     89     94    100    101    204    104    106    110
   115    116    121    122    128    129    135    137    139    140    143
   113    112    120    122    378    162    135    137    139    138    143
   147    151    193    219    223    232
   147    149    192    218    221    231
```

```
biogrid_statistics
complexes_statistics
zhu_paper_statistics
low_thruput_statistics
p53_statistics
```

7 Graphical functions

So far, **iRefR** contains three functions related to the graph of the network. **iRefR** makes use of the **graph**, **igraph** and **RBGL** packages as dependencies, so many graph-theoretical related functions may be implemented by the user. The default graphical package is **igraph** but the user can specify if he/she wants to work with **graph**.

convert_edgeList_to_graph and **convert_graph_to_edgeList** perform the

format conversion between an iRefIndex/edgeList table and a graph object. **summary_graph** offers some basic information on the given graph.

7.1 convert_edgeList_to_graph

```
> ## undirected case:
> graph_binary_INTACT = convert_edgeList_to_graph(edgeList_binary_INTACT,
+  "undirected", "igraph")
> graph_complex_INTACT_s = convert_edgeList_to_graph(
+ edgeList_complex_INTACT_s)
> graph_complex_INTACT_m = convert_edgeList_to_graph(
+ edgeList_complex_INTACT_m)
> graph_all_INTACT =
+  convert_edgeList_to_graph(edgeList_all_INTACT)
> summary(graph_binary_INTACT)

Vertices: 193
Edges: 123
Directed: FALSE
No graph attributes.
Vertex attributes: name.
Edge attributes: weight.

> summary(graph_complex_INTACT_s)

Vertices: 392
Edges: 834
Directed: FALSE
No graph attributes.
Vertex attributes: name.
Edge attributes: weight.

> summary(graph_complex_INTACT_m)

Vertices: 392
Edges: 5636
Directed: FALSE
No graph attributes.
Vertex attributes: name.
Edge attributes: weight.

> summary(graph_all_INTACT)

Vertices: 542
Edges: 951
Directed: FALSE
No graph attributes.
Vertex attributes: name.
Edge attributes: weight.

> ## directed case:
> graph_binary_INTACT_dir = convert_edgeList_to_graph(
```

```
+ edgeList_binary_INTACT_dir, "directed")
> graph_complex_INTACT_sdir =
+  convert_edgeList_to_graph(
+ edgeList_complex_INTACT_sdir, "directed")
> summary(graph_binary_INTACT_dir)

Vertices: 156
Edges: 108
Directed: TRUE
No graph attributes.
Vertex attributes: name.
Edge attributes: weight.

> summary(graph_complex_INTACT_sdir)

Vertices: 392
Edges: 849
Directed: TRUE
No graph attributes.
Vertex attributes: name.
Edge attributes: weight.
```

In the undirected case, it can be observed that the sum of binary and complex nodes/vertices and binary and complex edges is not equal to the number of nodes/vertices and edges in the complete database (all_INTACT). This is due to the existence of polymer interactions, repeated nodes between both lists or repeated edges between both lists. Comparing the undirected and the directed cases, it can be observed that, for the binary case, the edge number is smaller than the undirected case because only interactions with bait information are used. For the complex spoke case, the edge number is bigger than the undirected case because edges with two baits give raise to two directed edges and only one undirected.

After converting the edgeLists to igraph-graphs, we can use the functions of the **igraph** package to study their graph properties. The following example gives the names of all nodes, the names of the nodes with degree equal to 1, one plot with all six graphs, and one plot with the actual degree distribution of the binary graph and its corresponding log-log representation.

```
V(graph_binary_INTACT)$name
V(graph_binary_INTACT)[degree(graph_binary_INTACT) == 1]$name

> par(mfrow=c(2,3))
> plot(graph_binary_INTACT, layout=layout.fruchterman.reingold,
+  vertex.size=3, vertex.color="green", frame=TRUE,
+  main="Binary", vertex.label=NA)
> plot(graph_complex_INTACT_s, layout=layout.fruchterman.reingold,
+  vertex.size=3, vertex.color="green", frame=TRUE,
+  main="Complex Spoke", vertex.label=NA)
> plot(graph_complex_INTACT_m, layout=layout.fruchterman.reingold,
+  vertex.size=3, vertex.color="green", frame=TRUE,
+  main="Complex Matrix", vertex.label=NA)
```

```
> plot(graph_all_INTACT, layout=layout.fruchterman.reingold,
+  vertex.size=3, vertex.color="green", frame=TRUE,
+  main="Binary and Complex Spoke", vertex.label=NA)
> plot(graph_binary_INTACT_dir, layout=layout.fruchterman.reingold,
+  vertex.size=3, edge.arrow.size=0.3, vertex.color="green",
+  frame=TRUE, main="Binary Directed", vertex.label=NA)
> plot(graph_complex_INTACT_sdir,
+  layout=layout.fruchterman.reingold, vertex.size=3,
+  edge.arrow.size=0.3, vertex.color="green", frame=TRUE,
+  main="Complex Spoke Directed", vertex.label=NA)
```

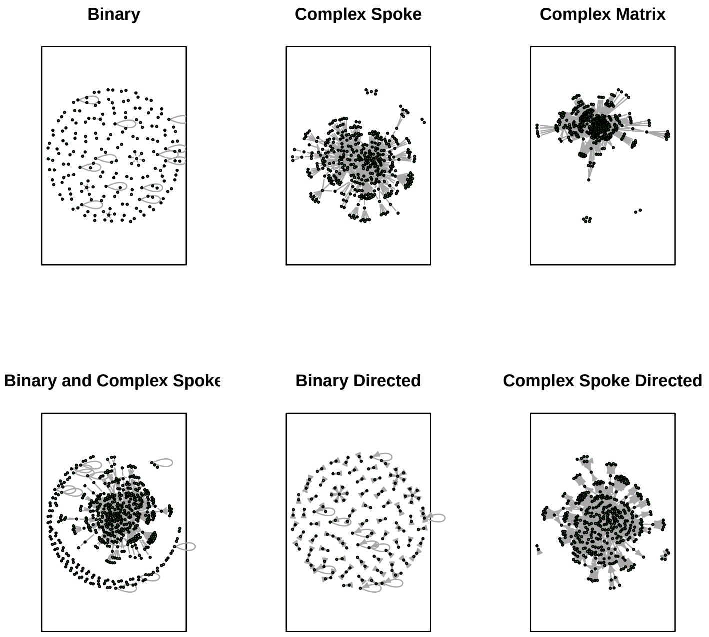


```
par(mfrow=c(1,2))
plot(degree.distribution(graph_binary_INTACT),
 main="Degree Distribution (Binary, INTACT, E.coli)")
plot(degree.distribution(graph_binary_INTACT),log="xy",
 main="Log-Log Degree Distribution (Binary, INTACT, E.coli)")
```

7.2 convert_edgeList_to_graph -Alternative Exercise

We can also use **graph** instead of **igraph** and several other analysis are possible. After converting the edgeLists to graph-graphs, we can use the functions of the **graph** package to study their graph properties. For example, the summary of number of nodes and edges, the list of nodes, the number of nodes without edges, the name of the node with most edges, the adjacent nodes to a given node and the accesibility of a node to other nodes.

```
graph_bin_INTACT = convert_edgeList_to_graph(
```

```
 edgeList_binary_INTACT, "undirected", "graph")
graph_com_INTACT_s = convert_edgeList_to_graph(
 edgeList_complex_INTACT_s, "undirected", "graph")
graph_com_INTACT_m = convert_edgeList_to_graph(
 edgeList_complex_INTACT_m, "undirected", "graph")

graph_bin_INTACT
graph_com_INTACT_s
graph_com_INTACT_m
nodes(graph_bin_INTACT)
numNoEdges(graph_bin_INTACT)
mostEdges(graph_bin_INTACT)
adj(graph_bin_INTACT,"875345")
acc(graph_bin_INTACT, "875345")
```

As the directed and undirected edgeLists do not have the same size, for the reasons explained above (using only information with bait and bidirectional edges), the number of nodes and edges are different between the undirected and the directed case.

A last application of this function might be finding all edges in a complex representation that have been confirmed with binary interactions, in case that a user wants to know how many binary interactions are known and reported between the members of a given protein complex. That might give us an idea of either false edges or edges that need to be confirmed. In this example, 11 edges of a matrix model of all complexes of the `INTACT` database happened to be confirmed by binary interactions from the same database.

```
graph_complex_confirmed_by_binaries_INTACT = intersection(
graph_bin_INTACT, graph_com_INTACT_m)
```

7.3 convert_graph_to_edgeList

```
> reconstructed_edgeList_binary_INTACT =
+  convert_graph_to_edgeList(graph_binary_INTACT)
> reconstructed_edgeList_binary_INTACT_dir =
+  convert_graph_to_edgeList(graph_binary_INTACT_dir,
+  "directed")
> reconstructed_edgeList_complex_INTACT_s =
+  convert_graph_to_edgeList(graph_complex_INTACT_s)
> reconstructed_edgeList_complex_INTACT_sdir =
+  convert_graph_to_edgeList(graph_complex_INTACT_sdir,
+  "directed")
```

In this exercise, we have reconstructed four of the edgeLists from their corresponding directed or undirected graphs. After comparing the reconstructed lists to the original ones, it can be observed that the format conversions have been succesful.

```
> setequal(dim(edgeList_binary_INTACT),
+
+  dim(reconstructed_edgeList_binary_INTACT))
```

```
[1] TRUE

> setequal(dim(edgeList_binary_INTACT_dir),
+
+  dim(reconstructed_edgeList_binary_INTACT_dir))

[1] TRUE

> setequal(dim(edgeList_complex_INTACT_s),
+
+  dim(reconstructed_edgeList_complex_INTACT_s))

[1] TRUE

> setequal(dim(edgeList_complex_INTACT_sdir),
+
+  dim(reconstructed_edgeList_complex_INTACT_sdir))

[1] TRUE
```

All four tests give TRUE when using **igraph** while give FALSE when using **graph** due to the fact that polymer information (loop edges) is not supported in the iRefR/graph option. The example can take 2 seconds for E.coli and up to 8' for human.

7.4 summary_graph

```
> summary = summary_graph(graph_all_INTACT, "igraph")

Vertices: 542
Edges: 951
Directed: FALSE
No graph attributes.
Vertex attributes: name.
Edge attributes: weight.
```

The function generates a list with information on nodes and edges, degree distribution and connected components. Histograms of degree distribution and nodes per connected component are generated, together with a plot of the graph.

```
summary$nodes_and_edges
table(summary$degree_distribution)
x11(); hist(summary$degree_distribution)

table(summary$nodes_per_connected_component)
x11(); barplot(summary$nodes_per_connected_component,
 xlab="Size Components", ylab="Number Components")
title(main="Distribution of Connected Components")

summary$graph_per_module[[1]]
```

The previous information can also be obtained using the **graph** package, with exception of the graph plots, which would need installation and use of the **Rgraphviz** package.

8 Bugs and Suggestions

Have you found any bug? Is there any particular analysis you would like to perform using this package? Please send your comments to: `i.m.donaldson@biotek.uio.no`

A Software needed to install packages from source using Windows

The easiest way to do it is the following:

1. Go to http://www.murdoch-sutherland.com/Rtools/ and install Rtools213.exe (this contains R toolset, Cygwin DLLs, MinGW compilers and tools, and some extras).
2. Go to http://www.miktex.org/, download and install the MiKTeX LaTeX generator.
3. Go to http://msdn.microsoft.com/en-us/library/ms669985.aspx, download and install the Microsoft HTML Help Workshop.
4. Go to http://www.jrsoftware.org/, download and install the Inno Setup self-installing package.
5. Check your environmental variables (right click on ”My PC”, then system properties, set environmental variables): There must be a path to the R-2.13.0/bin directory and a new variable R_LIBS must be set to the R-2.13.0/library directory.

B Code for selecting only human-human or E.coli-E.coli interactions

Currently, the easiest way to do it is the following:

```
> human_human_list = data.frame(irefindex_curr_human$taxa,
+  irefindex_curr_human$taxb)
> tmp = do.call(`paste`, c(unname(human_human_list),
+  list(sep=".")))
> irefindex_curr_human = irefindex_curr_human[
+  tmp == "taxid:9606(Homo sapiens).taxid:9606(Homo sapiens)" |
+  tmp == "-.taxid:9606(Homo sapiens)",]
```
